# Exploring the relation between the EQ-5D-5L pain/discomfort and pain and itching in a sample of burn patients

**DOI:** 10.1186/s12955-020-01394-0

**Published:** 2020-05-19

**Authors:** I. Spronk, G. J. Bonsel, S. Polinder, M. E. van Baar, M. F. Janssen, J. A. Haagsma

**Affiliations:** 1 Department of Public Health, Erasmus MC, University Medical Center Rotterdam, P.O. Box 2040, 3000 CA Rotterdam, The Netherlands; 2 Association of Dutch Burn Centres, Maasstad Hospital, Rotterdam, the Netherlands; 3 EuroQol Group Executive Office, Rotterdam, The Netherlands; 4 Section Medical Psychology and Psychotherapy, Department of Psychiatry, Erasmus MC, Rotterdam, The Netherlands

**Keywords:** Health-related quality of life, EQ-5D-5L, Pain, Itching, POSAS, Burn patients

## Abstract

**Background:**

The EQ-5D domain pain/discomfort (PD) uses one item to capture pain and other aspects of discomfort, like itching. This study explored how pain, itching and the EQ-5D-5L PD domain relate to each other in a sample of burn patients.

**Methods:**

Adult burn patients completed the EQ-5D-5L and the Patient and Observer Scar Assessment Scale (POSAS) 5–7 years after sustaining their injury. The POSAS includes a separate pain and an itching item. Spearman’s correlation coefficient established the association between the EQ-5D-5L PD and the POSAS pain and itching item. With multivariable regression analysis the linear association between the POSAS pain and itching item and EQ-5D-5L PD domain was tested.

**Results:**

Data from 245 patients were included. Mean EQ-5D-5L index value was 0.87 and 39.2% reported at least slight problems on the EQ-5D-5L PD domain. Most patients gave corresponding answers on the EQ-5D-5L PD domain and on the POSAS pain (73%) and itching (70%) item. Spearman correlation coefficients of the EQ-5D-5L PD domain with the POSAS pain and itching were 0.468 (*p* < 0.001) and 0.473 (*p* < 0.001), respectively. Among respondents with pain and without itching and respondents with itching and without pain, Spearman correlation coefficients were 0.585 (*p* = 0.076) and 0.408 (*p* = 0.001), respectively. POSAS pain (unstandardized Beta = 0.14) and POSAS itching (unstandardized Beta = 0.08) were significantly associated with EQ-5D-5L PD domain (*p* < 0.001).

**Conclusions:**

Our findings indicate that, in a sample of burn patients, pain and itching are captured by the broader EQ-5D-5L PD domain. The EQ-5D-5L PD domain can thus be used to assess pain and itching in relation to HRQL, but the POSAS pain and itching items are more sensitive. The EQ-5D-5L is, however, no replacement of the POSAS when the POSAS is used for its primary aim; assessment of scar quality.

**Trial registration:**

Netherlands Trial Register (NTR6407).

## Background

Health-related quality of life (HRQL) assessment is increasingly used to evaluate the consequences of a disease, condition or symptom, to assess the impact of health interventions on these consequences, and to investigate the quality of care [1–3]. An extensively-used instrument is the 5-dimensional EuroQol instrument (EQ-5D). This short self-report instrument has been validated for many diseases, and is available in many languages [4, 5]. The EQ-5D descriptive system consists of five domains: mobility, self-care, usual activities, pain/discomfort and anxiety/depression, each of which can be scored through a 3 or 5 level ordinal scale [6].

Pain, symptoms and other aspects of discomfort are captured by the EQ-5D domain ‘pain/discomfort’ (EQ-5D PD). However, unlike pain, other symptoms, conditions, or complaints, such as itching, nausea, or feeling breathless, are not explicitly defined. Still, these undefined aspects are assumed to be captured by this domain description. However, one study that explored the extent to which psoriasis symptoms/problems were captured by the PD challenged this assumption [5]. Supported by literature evidence, in-depth interviews with patients and clinicians, and psychometric analyses of existing sources, the ‘bolt-on’ domain ‘skin irritation (e.g. itching)’ was therefore added to the EQ-5D to create a psoriasis-specific version of the EQ-5D.

We studied whether itching and pain are (sufficiently) captured by the EQ-5D-5L PD domain, using EQ-5D-5L data as well as separate pain and itching data from burn patients, a specific patient group that experiences pain and/or itching. Chronic pain and chronic itching in various degrees of intensity and frequency are common problems among burn patients [7–9]. Pain prevalence is as high as 92% during hospitalization and decreases towards 48% at 6 months, and 42% at 12 months following burns [7]. After 4–7 years, still 26% of burn patients experience non-trivial, disturbing pain, with 12% reporting high pain scores [10]. The prevalence rate for itching is as high as 87% at 3 months post burn and drops to 67% 2 years following burns [8]. Itching remains a prevalent problem (44–49%) in the long-term; 25% even reported severe itching [10–12].

Our study aimed to explore how pain, itching and the EQ-5D-5L PD domain relate to each other. First, we investigated how pain and itching, as measured separately through items of the Patient and Observer Scar Assessment Scale (POSAS), relate to the response to the EQ-5D-5L PD. Second, we investigated the association, if any, between the POSAS pain and itching items and the remaining EQ-5D-5L domains. Third, we investigated if variability in pain and itching scores, measured with the POSAS, was captured by the EQ-5D-5L PD domain.

## Methods

### Participants

The study had a cross-sectional design. Participants included adult burn patients with a hospital stay of ≥1 day or who had surgical treatment in one of the dedicated Dutch burn centres (Red Cross Hospital Beverwijk, Martini Hospital Groningen, Maasstad Hospital Rotterdam) between August 2011 and September 2012 [13]. This sample was extended with patients with severe burns (> 20% total body surface area (TBSA) burned in patients ≤50 years old; > 10% TBSA burned in patients > 50 years; or TBSA full thickness > 5% (based on the criteria of the American Burn Association [14]) admitted between January 2010 and March 2013. Those with pre-existing cognitive impairments and with insufficient knowledge of the Dutch language were excluded.

Between March 2017 and March 2018, eligible patients were invited to participate via a postal invitation, including an information letter, an informed consent form and the survey. Patients who did not respond within 3 weeks received a phone call (or a postal reminder when there was no telephone number available) to discuss participation. The study was performed according to the principles of the Declaration of Helsinki, registered at the Netherlands Trial Register (NTR6407), and approved by the Ethics Committee (registration number NL59981).

### Measures

Background information was derived from the Dutch Burn Repository R3 [15]. Patient characteristics consisted of age, gender and having chronic diseases. The presence of any pre-existing chronic disease was defined as having comorbidity; patients without a pre-existing chronic disease were defined as not having comorbidity. Injury characteristics, related to the entire burn history, consisted of percentage total body surface area (%TBSA) burned, % full-thickness burns, etiology, date of injury, number of surgeries for burns, length of hospital stay for burn injury, reconstructive surgery, artificial ventilation, and time since burn. The survey included two existing questionnaires: the EQ-5D-5L (Dutch language version) and the patient part of the Patient and Observer Scar Assessment Scale (POSAS) (Dutch language version), all paper and pencil self-assessment.

The EQ-5D-5L includes items on five domains: mobility, self-care, usual activities, pain/discomfort, and anxiety/depression. Each domain has five ordered response categories: no problems, slight problems, moderate problems, severe problems and extreme problems. An EQ-5D-5L index value was calculated ranging from 0 (death) to 1 (full health) based on the answers of the five domains using the Dutch EQ-5D-5L value set [16, 17]. The EQ-5D-5L measure also consists of a visual analogue scale (VAS) for general health that ranges from 0 (worst imaginable health) to 100 (best imaginable health) [18].

Itching and pain were separately measured by the POSAS 2.0 (patient part) [19]. The POSAS consists of six items: pain, itching, colour, thickness, relief and pliability of a scar [20]. For present study, only the two items on pain and itching were used. Items were scored on a 10-point VAS scale ranging from 1 (no pain/itch) to 10 (extreme pain/itch). Participants were asked to complete these POSAS items for their – in their opinion – most severe scar.

### Data analyses

IBM SPSS Statistics 23 was used for the analyses. A sample size calculation was performed. A sample size of 118 patients was required when alpha was set at 0.05 and a power of 80%. This enables the detection of *R*^*2*^ ≥ 0.15 (G-power was used to calculate the sample size with a given power and effect size). A non-response analysis was performed to study whether responders and non-responders differed. Mann Whitney U tests were used for continuous variables and chi-square tests for categorical variables. Descriptive statistics were used to assess characteristics as well as outcomes of the EQ-5D-5L and the POSAS pain and itching items. Generally, results were studied overall and for the subgroups: patients with versus without comorbidity, and the subgroups patients with pain and no itching (as reported on the POSAS) and patients with itching and no pain (POSAS).

Head-to-head comparisons were used to compare outcomes of the EQ-5D-5L PD domain and the POSAS pain and itching items. The proportion of patients with corresponding answers was assessed and tested using a chi-squared test. Corresponding answers were defined as reporting problems on both the EQ-5D-5L PD domain and a POSAS item, so for example for the EQ-5D-5L PD domain and POSAS pain, a patient reported corresponding answers as he/she reported problems on both EQ-5D-5L PD domain (score > 1) and POSAS pain (score > 1). To assess whether the EQ-5D-5L PD domain reflects the worst level of both POSAS items, a POSAS worst count variable was created based on the POSAS pain and itching items. So, if POSAS pain was scored higher (worse) than POSAS itching, this score was used in the worst level variable, or vice versa. The worst outcome was compared with the EQ-5D-5L PD domain outcome.

We used the Spearman rank correlation coefficient to establish convergent validity of the EQ-5D-5L PD using the POSAS items as reference. Rank order correlation between the EQ-5D-5L PD domain and POSAS pain and itching items, as well as between all other EQ-5D-5L domains and POSAS pain and itching items were studied, both in the total sample as in the defined subgroups [21]. Both the EQ-5D-5L PD domain and the POSAS items were treated as numerical variables. Also, the rank order correlation between the EQ-5D-5L PD domain and the POSAS worst count variable was assessed, both in the total sample as in patients that reported both pain and itching on the POSAS. According to Cohen’s criteria, strength of the correlations was regarded strong if *r* ≥ 0.50, moderate if *r* ≥ 0.30–0.49, and weak if *r* ≥ 0.10–0.29 [22].

Then, it was assessed to what extent variability in pain and itching was captured by the EQ-5D-5L PD domain. The correlation between the POSAS pain and itching item and the correlation with the EQ-5D-5L PD domain was studied. For each combination of POSAS pain and itching outcomes, the mean EQ-5D-5L PD domain score was calculated. Multivariable regression analyses were applied to predict EQ-5D-5L PD domain from the POSAS pain and itching items, with relevant demographic (age, sex, comorbidity) and clinical variables (length of hospital stay, %TBSA burned, number of surgeries, aetiology) added. Lastly, the EQ-VAS was predicted by the POSAS pain and itching items, as well as by the EQ-5D-5L PD. The significance level for all analyses was set at *p* < 0.05.

### Hypotheses

1. Itching is captured by the EQ-5D-5L PD, which is tested by the presence of problems on the EQ-5D-5L PD domain for the subgroup of patients who do not score problems on POSAS pain, but do score problems on POSAS itching.
2. The association between the EQ-5D-5L PD domain scores and the POSAS pain item score is of comparable magnitude compared to the association between the EQ-5D-5L PD domain and the POSAS itching item score. If both symptoms coincide, the EQ-5D-5L PD domain reflects the worst level of both POSAS items.
3. The EQ-5D-5L PD domain score is stronger related to the POSAS pain item score in patients without rather than with comorbidity, because patients with comorbidity have a higher probability to experience pain due to their comorbid chronic disease.
4. The association between the other EQ-5D-5L domains and the POSAS pain and itching items is lower compared to that between EQ-5D-5L PD domain and the same items.
5. There is a strong correlation between the EQ-5D-5L PD domain score and the POSAS pain and itching item score.

## Results

### Participants

A total of 517 patients were eligible, of whom 257 were willing to participate. Two hundred forty-five patients (47.4%) completed all items of the EQ-5D-5L and POSAS and were included in this study. Non-response analysis showed that responders were more often females (*p* = 0.03) and were older (*p* < 0.01) than non-responders (Additional file 1). Table 1 shows the characteristics of the study population. Participants had a mean age of 47.4 years (SD 16.8) and 62.0% was male. Median %TBSA burned was 6.0% (IQR: 2.0–12.5) and mean length of stay 17.9 days (SD 22.2). Patients with comorbidity were significantly older, more often female, had more severe burns, a longer hospital stay and underwent more surgeries.

**Table 1:** Characteristics of study population

Variable	Total sample (***n*** = 245)	Without comorbidity (***n*** = 183)	With comorbidity (***n*** = 62)	***P***-value
Gender: male, n(%)	152 (62.0%)	124 (67.8%)	28 (45.2%)	0.002
Age at follow-up (M, SD)	47.4 (16.8)	38.5 (16.3)	57.2 (14.2)	< 0.001
%TBSA (Median, IQR)	6.0 (2.0–12.5)	5.0 (1.5–11.0)	8.0 (3.0–16.5)	0.012
Length of hospital stay (M, SD)	17.9 (22.2)	15.6 (20.8)	24.9 (24.7)	< 0.001
Number of surgeries (M, SD)	1.3 (2.0)	1.1 (1.9)	1.8 (2.2)	0.006
Aetiology, n(%)				0.197
Flame	141 (58.0%)	101 (55.5%)	40 (65.6%)	
Scald	46 (19.0%)	34 (18.6%)	12 (19.7%)	
Other	56 (23.0%)	47 (25.8%)	9 (14.8%)	
Time since burn (years) (M, SD)	5.6 (0.5)	5.6 (0.5)	5.6 (0.5)	0.768
Comorbidity, n pre-existing chronic conditions (%)				
No	183 (74.7%)			
One	44 (18.0%)			
More than one	18 (7.3%)			
**EQ-5D-5L scores**				
Index value (M, SD)	0.87 (0.18)	0.90 (0.09)	0.77 (0.23)	< 0.001
EQ-VAS (M, SD)	81.6 (15.7)	84.1 (14.6)	74.2 (16.5)	< 0.001
Mobility (% with problems)	15.5%	9.8%	32.3%	< 0.001
Self-care (% with problems)	7.8%	3.8%	19.4%	< 0.001
Usual activities (% with problems)	23.3%	16.9%	41.9%	0.001
Pain/discomfort (% with problems)	39.2%	33.3%	56.5%	0.001
Anxiety/depression (% with problems)	31.4%	27.3%	43.5%	0.009
**POSAS Patient Scale scores**				
% patients with pain (POSAS pain score ≥ 2)	25.7%	22.4%	35.5%	0.174
% patients with itching (POSAS itching score ≥ 2)	48.2%	48.6%	46.8%	0.166

*M, SD* mean, standard deviation, *IQR* interquartile range, *%TBSA* percentage total body surface area, *EQ-VAS* EQ Visual Analogue Scale

### EQ-5D-5L and POSAS outcomes

Mean EQ-5D-5L index value was 0.87 (SD 0.18) and the mean EQ-VAS was 81.6 (SD 15.7) (Table 1). Most problems were reported on the PD domain; 39.2% of the participants reported at least mild problems. In total, 63 respondents (25.7%) reported pain (POSAS pain score ≥ 2) and 118 (48.2%) reported itching (POSAS itching score ≥ 2). Patients with comorbidity had significantly worse outcomes on both the EQ-5D-5L as on the POSAS pain and itching items (Table 1).

### Head-to-head comparisons

#### EQ-5D-5L PD domain and POSAS pain

In total, 27% of the patients (*n* = 67) reported non-corresponding answers on the POSAS pain item and EQ-5D-5L PD domain (Table 2). The majority of respondents with non-corresponding answers (75%, *n* = 50) reported no pain on the POSAS and slight to severe problems on the EQ-5D-5L PD domain. Chi square test showed that answers on both items were related (X^2^ (1)=40.735; *p* < 0.001). For patients with comorbidity, the percentage of respondents with non-corresponding answers was 34%, of which most (81%) reported no pain on the POSAS and slight to severe problems on the EQ-5D-5L PD domain. For patients without comorbidity, the percentage of respondents with non-corresponding answers was 25%, with 72% of them reporting no pain on the POSAS and slight to severe problems on the EQ-5D-5L PD domain. Of the 10 respondents who reported pain and no itching (POSAS), 70% reported problems on the EQ-5D-5L PD domain. For respondents with and without comorbidity, this percentage was 67 and 81%, respectively.

**Table 2:** Head-to-head comparison of outcomes of the EQ-5D-5L pain/discomfort domain and POSAS pain and itching items

	EQ-5D-5L pain/discomfort	
	Number of patients that reported problems (n, %)	Number of patients that reported no problems (n, %)
**POSAS pain**		
Number of patients that reported pain (n, %)	**46 (18.8%)**	17 (6.9%)
Number of patients that reported no pain (n, %)	50 (20.4%)	**132 (53.9%)**
**POSAS itching**		
Number of patients that reported itching (n, %)	**70 (28.6%)**	48 (19.6%)
Number of patients that reported no itching (n, %)	26 (10.6%)	**101 (41.2%)**
**POSAS worst count variable**^**a**^		
Number of patients that reported pain/itching (n, %)	**77 (31.4%)**	51 (20.8%)
Number of patients that reported no pain/itching (n, %)	19 (7.8%)	**98 (40.0%)**

Values printed in bold are considered corresponding answers, whereas the values not printed in bolt are considered non-corresponding answers

^a^a POSAS worst count variable was created based on the POSAS pain and itching items. If POSAS pain was scored higher (worse) than POSAS itching, this score was used in the worst level variable, or vice versa

#### EQ-5D-5L PD domain and POSAS itching

Non-corresponding answers on the POSAS itching item and EQ-5D-5L PD domain were given by 30% of the patients (*n* = 74) (Table 2), of which 65% (*n* = 48) reported itching on the POSAS and no problems on the EQ-5D-5L PD domain. Chi square test showed that answers on both items were related (X^2^ (1)=38.741; *p* < 0.001). For patients with comorbidity, the percentage of respondents with non-corresponding answers was 31%, of which 33% reported itch on the POSAS and no on the EQ-5D-5L PD domain. For patients without comorbidity, 29% gave non-corresponding answers, with 75% of them reporting itch on the POSAS and no problems on the EQ-5D-5L PD domain. Of the 65 respondents who reported itching and no pain (POSAS), 48% reported problems on the EQ-5D-5L PD domain. For respondents with and without comorbidity, this percentage was 70 and 44%, respectively.

#### EQ-5D-5L PD domain and POSAS worst count variable

A total of 70 patients (29%) gave non-corresponding answers on the POSAS worst count variable versus the EQ-5D-5L PD domain. Most of them (73%) reported pain and/or itching on the POSAS and no pain/discomfort on the EQ-5D-5L PD domain. Comparing patients reporting no problems, 117 patients (48%) reported no problems on the combined POSAS variable, whereas 149 patients (61%) reported no problems on the EQ-5D-5L PD domain.

### Convergent validity of the EQ-5D-5L PD domain

#### EQ-5D-5L PD domain and POSAS pain and itching

Spearman rank correlation coefficients of the EQ-5D-5L domains and the POSAS pain and itching items are displayed in Table 3. The Spearman rank correlation coefficients of the EQ-5D-5L PD domain with the POSAS pain and POSAS itching were 0.468 (*p* < 0.001) and 0.473 (*p* < 0.001), respectively. In the subgroup of patients reporting pain but no itching on the POSAS, Spearman’s rank correlation coefficient was 0.585 (*p* = 0.076), indicating a strong correlation. In the subgroup of patients with itching and no pain, the correlation was 0.408 (*p* = 0.001), indicating a moderate correlation. The correlation between the EQ-5D-5L PD domain and the POSAS pain item and between the EQ-5D-5L PD domain and the POSAS itching item were comparable in respondents with and without comorbidity.

**Table 3:** Association of all EQ-5D-5L domains (5 level score) with POSAS pain and itching (1–10 score) in terms of Spearman’s rank correlation

		Other EQ-5D-5L domains			
	Pain/ discomfort	Mobility	Self-care	Usual activities	Anxiety/ depression
**All (*n*** **= 245)**					
POSAS pain	0.468^*^	0.153^*^	0.099	0.359^*^	0.174^*^
POSAS itch	0.473^*^	0.058	0.096	0.301^*^	0.192^*^
**Subgroup POSAS pain, no itching (*n*** **= 10)**					
POSAS pain	0.585	0.041	NA	0.208	0.609
**Subgroup POSAS itching, no pain (*n*** **= 65)**					
POSAS itch	0.408^*^	0.030	0.235	0.303^*^	0.256^*^
**Subgroup with comorbidity (*n*** **= 62)**					
POSAS pain	0.453^*^	0.019	0.106	0.272^*^	0.206
POSAS itch	0.472^*^	−0.025	0.141	0.235	0.243
**Subgroup without comorbidity (*n*** **= 183)**					
POSAS pain	0.451^*^	0.196^*^	0.047	0.374^*^	0.131
POSAS itch	0.481^*^	0.095	0.047	0.336^*^	0.174^*^

^*^p < 0.05 for the correlation, based on Spearman’s correlation coefficient

#### EQ-5D-5L PD domain and POSAS worst count variable

In the total sample, the rank correlation between the EQ-5D-5L PD domain and the combined worst count POSAS variable was 0.530 (*p* < 0.001).

#### EQ-5D-5L other domains and POSAS pain and itching

The correlation coefficients of the other EQ-5D-5L domains and the POSAS pain item ranged from 0.099 (*p* = 0.121) for self-care to 0.359 (*p* < 0.001) for usual activities (Table 3). The correlation coefficients of the other EQ-5D-5L domains and the POSAS itching item ranged from 0.058 (*p* = 0.368) for mobility to 0.301 (*p* < 0.001) for usual activities.

### Variability in pain and itching and the EQ-5D-5L PD domain

**Fig. 1:**
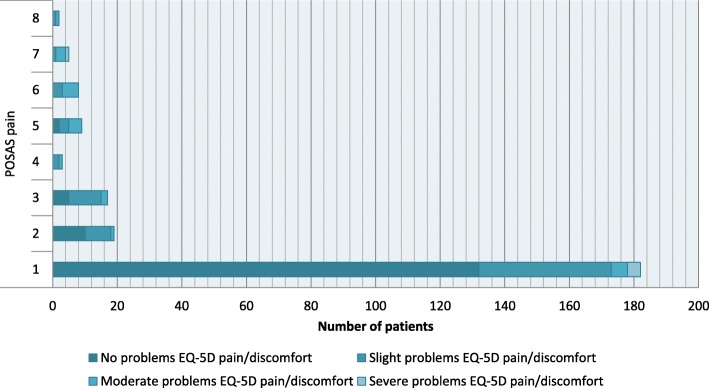
Outcomes of the EQ-5D-5L pain/discomfort domain versus POSAS pain outcomes. POSAS pain was scored on a 10-point scale ranging from 1 (no pain) to 10 (extreme pain)

**Fig. 2:**
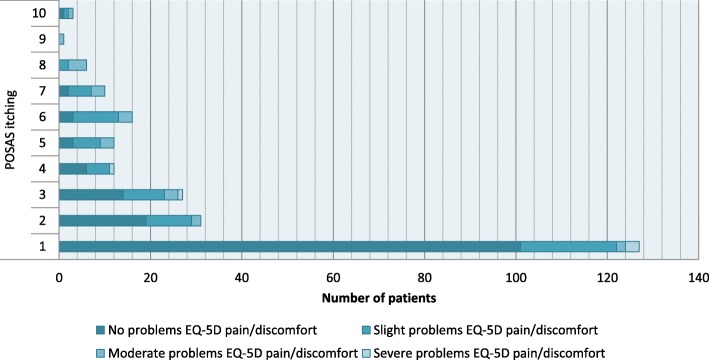
Outcomes of the EQ-5D-5L pain/discomfort domain versus POSAS itching outcomes. POSAS itching was scored on a 10-point scale ranging from 1 (no itching) to 10 (extreme itching)

**Fig. 3:**
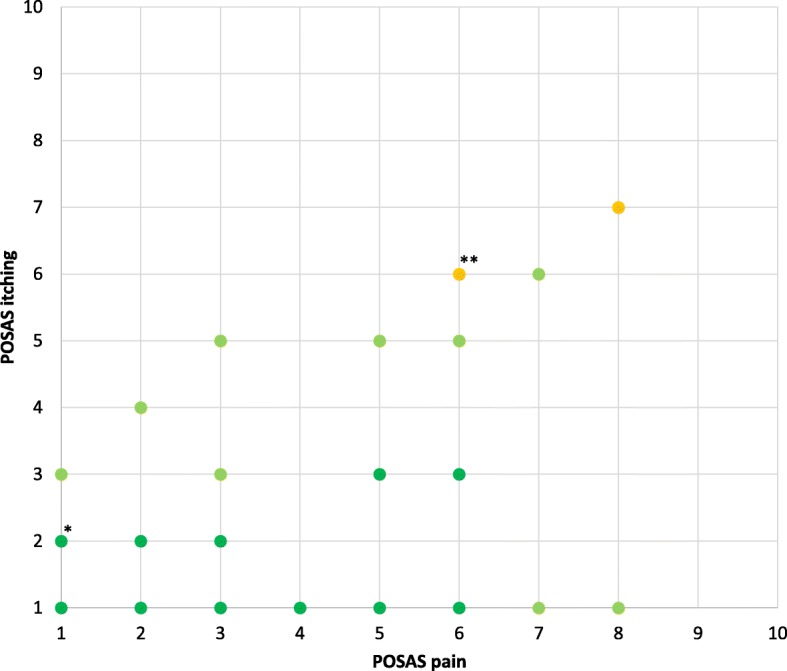
Mean EQ-5D-5L pain/discomfort (PD) domain scores for combinations of POSAS pain and itching outcomes that appear ≥2 times.The colored dots represent this mean EQ-5D-5L PD domain score, with dark green: mean EQ-5D-5L PD domain score = 1 - < 2; green: mean EQ-5D-5L PD domain score = 2 - < 3; orange: mean EQ-5D-5L PD domain score = 3 - < 4; red: mean EQ-5D-5L PD domain score = 4–5. *21 patients had a POSAS pain score of 1 (x-axis) and POSAS itching score of 2 (y-axis) and the mean EQ-5D-5L pain/discomfort domain score of these patients was 1.3 (shown as dark green spot).** 3 patients had a POSAS pain score of 6 (x-axis) and POSAS itching score of 6 (y-axis) and the mean EQ-5D-5L pain/discomfort domain score of these patients was 3.0 (shown as yellow spot)

Outcomes of the EQ-5D-5L PD domain versus POSAS pain outcomes are displayed in Fig. 1, and versus POSAS itching outcomes in Fig. 2. The correlation between the POSAS pain and itching items and the correlation with the EQ-5D-5L PD domain is shown in Fig. 3, with the Spearman rank correlation coefficient between the POSAS pain and itching items being 0.489 (*p* < 0.001), indicating a moderate correlation between both POSAS items.

Multivariate regression analysis showed that POSAS pain (unstandardized Beta = 0.14) and POSAS itching (unstandardized Beta = 0.08) were significantly associated with the EQ-5D-5L PD domain (both *p* < 0.001).

In respondents with comorbidity, POSAS pain (unstandardized Beta = 0.15; *p* = 0.040), but not POSAS itching (unstandardized Beta = 0.08; *p* = 0.155) was significantly associated with the EQ-5D-5L PD domain. In respondents without comorbidity, however, both POSAS items pain (unstandardized Beta = 0.14; *p* < 0.001) and itching (unstandardized Beta = 0.09; *p* < 0.001) were significantly associated with the EQ-5D-5L PD domain.

Prediction of the EQ-VAS was also studied using multivariate regression analysis. POSAS pain (unstandardized Beta = 0.61) and POSAS itching (unstandardized Beta = − 0.16) were not significantly associated (*p* > 0.05) with the EQ-VAS, whereas the EQ-5D-5L PD domain was found to be associated (unstandardized Beta = − 7.94; *p* < 0.001) (Additional file 2). Also, age and having comorbidity were found to be significant predictive factors for the EQ-VAS (Additional file 2). In the subgroups of patients with and without comorbidity, similar results were found with only the EQ-5D-5L PD domain being significantly associated with the EQ-VAS.

## Discussion

This study investigated the sensitivity of the EQ-5D-5L PD domain for pain and itching in a sample of burn patients. Overall, most patients gave corresponding answers on the EQ-5D-5L PD domain and on the POSAS pain and itching item, but details were different. Overall, more patients reported problems when assessed by the POSAS compared to the EQ-5D-5L PD domain. Both POSAS pain and EQ-5D-5L PD domain, and POSAS itching and EQ-5D-5L PD domain were shown to be moderately correlated, with similar associations in patients with and without comorbidity. EQ-5D-5L usual activities, anxiety/depression and mobility were to a lesser extent correlated to the POSAS pain and/or itching item. A strong correlation between EQ-5D-5L PD domain and POSAS pain in patients with pain was found, a moderate correlation between EQ-5D-5L PD domain and POSAS itching in patients reporting itching was found. Scale congruence is present between the EQ-5D-5L PD domain score and the POSAS pain and itching item score.

Our results, which indicate that more problems are reported by the use of a single item question compared to a composite measure, are in line with an earlier study. The study by Tsuchiya et al. examined splitting of the composite EQ-5D-5L PD dimension into separate items and found that problems were more frequently reported by the separate items (49% of sample reporting problems) compared to the composite dimension (43% of sample reporting problems), however, this difference was not statistically significant [23]. Interestingly, this difference was larger and statistically significant for the anxiety/depression dimension in the same study. The proportion of patients that reported problems for the separate items (55%) was substantially larger than for the composite anxiety/depression dimension (43%). The authors suggested that the PD dimension is interpreted more literally (read as pain or discomfort) compared to the anxiety/depression dimension (possibly read as anxiety and depression) [23].

Our results confirm our first hypothesis that the correlation between the EQ-5D-5L PD domain and the POSAS pain item is comparable to the correlation between the EQ-5D-5L PD domain and the POSAS itching item. Only a small percentage of patients reported non-corresponding answers, e.g. no problems on the EQ-5D-5L PD domain (score = 1), and problems on the POSAS pain (score > 1). Part of this inconsistency may be the consequence of differential instruction: the POSAS was completed for the patients –in their opinion– worst scar. The EQ-5D-5L PD domain on the other hand, reflects the patient’s overall condition. Most patients (75%) that reported inconsistently outcomes, reported pain in the EQ-5D-5L PD domain and yet no pain on the POSAS pain item. A possible explanation for this finding may be that pain outside the scar may have a relation to a pre-existing condition (comorbidity). However, the correlation between the EQ-5D-5L PD domain and the POSAS pain item was higher rather than lower in the group patients without comorbidity than in the group with comorbidity. For itching, a different pattern was observed; most patients with inconsistent answers reported itching on the POSAS itching item, but no PD in the EQ-5D PD domain. Potentially because these patients did not consider itching as discomfort, because of fluctuations of itching levels, or because they simply overlook the discomfort addition.

Subgroup analysis, in patients with respectively pain and itching, showed that EQ-5D-5L PD domain and POSAS pain had a good correlation, whereas the EQ-5D-5L PD domain and POSAS itching showed a moderate correlation. Pain seems thus to be sufficiently covered by the EQ-5D-5L PD; however, results on itching are less conclusive in our sample of burn patients. An earlier study in psoriasis patients identified ‘skin irritation, including itching’ as a separate candidate domain. Psychometric analyses showed that the new domain captured additional variance over the existing five EQ-5D-5L domains [5]. Additional analyses on both the explanatory and discriminatory power of an additional itching domain are valuable to conclude whether an extra domain captures additional information.

This study has strengths and limitations. A strength was the unselected cohort nature, and the high prevalence of pain and itching which allowed us to study whether itching and pain are captured by the EQ-5D-5L PD domain, including interaction effects. Another strength was that both instruments were validated in the burn population [20, 24]. A limitation was the relatively low variability in pain and itching reported by the patients, only few patients reported severe pain and/or itching, potentially due to the long-term follow-up data that we used - this limits scale correspondence to be scrutinized. Another apparent limitation was the difference in instruction (timeframe, body part to focus on). The EQ-5D-5L refers to your health today, whereas the POSAS asks about the past weeks. By using a different timeframe, answers might be slightly different on the two instruments. Also, burn scars can cause other problems that are measured by the POSAS instrument, including pliability and thickness of a scar. These items might also be detected by the EQ-5D-5L PD domain. This was, however, beyond the scope of present study to investigate this, but it might be an interesting topic for future research.

## Conclusions

Our findings indicate that, in a sample of burn patients, pain and itching are captured by the broader EQ-5D-5L PD domain. The EQ-5D-5L PD domain can thus be used to assess pain and itching in relation to HRQL, but the POSAS pain and itching items are more sensitive. The EQ-5D-5L is, however, no replacement of the POSAS when the POSAS is used for its primary aim; assessment of scar quality.

## Supplementary information

**Additional file 1.** Characteristics of adult responders versus non-responders.

**Additional file 2.** Multivariate model for the EQ-VAS, including EQ-5D-5L pain/discomfort domain, POSAS pain and itching item, and relevant demographic and clinical factors.

## Data Availability

The dataset used and analysed during the current study are available from the corresponding author on reasonable request.

## Abbreviations

EQ-5D: 5-dimensional EuroQol instrument
HRQL: Health-related quality of life
PD: Pain/discomfort
POSAS: Patient and Observer Scar Assessment Scale
TBSA: Total body surface area
VAS: Visual analogue scale
