# Supplementary material for: Exploring the relation between the EQ-5D-5L pain/discomfort and pain and itching in a sample of burn patients
Source: Health Qual Life Outcomes. 2020 May 19;18:144. doi: 10.1186/s12955-020-01394-0 (PMC7236121; doi:10.1186/s12955-020-01394-0)
Supplement: Supplementary file 1 — Additional file 1. Characteristics of adult responders versus non-responders. [file 12955_2020_1394_MOESM1_ESM.docx]

**Appendix 1. Characteristics of adult responders versus non-responders**

| **Variable** | **Responders (n=245)** | **Non-responders (n=261)** | **p-value** |
| --- | --- | --- | --- |
| **Gender:** Male, n(%) | 152 (62.0%) | 193 (71.0%) | 0.032 |
| **Age at burn (M, SD)** | 41.8 (16.8) | 38.4 (17.5) | 0.009 |
| **%TBSA burned (M, SD)** | 9.8 (12.4) | 9.4 (13.7) | 0.180 |
| **%TBSA full-thickness (M, SD)** | 3.8 (8.5) | 3.9 (9.4) | 0.300 |
| **Length of hospital stay (M, SD)** | 17.9 (22.2) | 16.9 (26.7) | 0.081 |
| **Nr of surgeries (M, SD)** | 1.3 (2.0) | 1.2 (2.4) | 0.055 |
| **Nr surgery,** **n(%)** |  |  |  |
| 0 | 89 (36.3%) | 128 (47.1%) |  |
| 1 | 109 (44.5%) | 93 (34.2%) |  |
| >1 | 47 (19.2%) | 51 (18.7%) |  |
| **Reconstructive surgery, n(%)** | 28 (11.4%) | 19 (7.0%) | 0.083 |
|  |  |  |  |
| **Mechanical ventilation, n(%)** | 39 (15.9%) | 44 (16.2%) | 0.782 |
|  |  |  |  |
| **Aetiology (%)** |  |  | 0.675 |
| Flame | 141 (58.0%) | 167 (61.9%) |  |
| Scald | 46 (19.0%) | 47 (17.4%) |  |
| Other | 56 (23.0%) | 56 (20.7%) |  |
