# Supplementary material for: Exploring the relation between the EQ-5D-5L pain/discomfort and pain and itching in a sample of burn patients
Source: Health Qual Life Outcomes. 2020 May 19;18:144. doi: 10.1186/s12955-020-01394-0 (PMC7236121; doi:10.1186/s12955-020-01394-0)
Supplement: Supplementary file 2 — Additional file 2. Multivariate model for the EQ-VAS, including EQ-5D-5L pain/discomfort domain, POSAS pain and itching item, and relevant demographic and clinical factors. [file 12955_2020_1394_MOESM2_ESM.docx]

**Appendix 2. Multivariate model for the EQ-VAS, including EQ-5D-5L pain/discomfort domain, POSAS pain and itching item, and relevant demographic and clinical factors**

| **EQ-5D-5L dimension** | **Unstandardized B** | **p-value** |
| --- | --- | --- |
| Constant | 100.870 | <0.001 |
| Age | -0.123 | 0.031 |
| EQ-5D-5L pain/discomfort domain | -7.940 | <0.001 |
| Comorbidity | -4.060 | 0.011 |
| F value  R-square | 24.3  0.234 | <0.001 |
